# SoilCam: A Fully Automated Minirhizotron using Multispectral Imaging for Root Activity Monitoring

**DOI:** 10.3390/s20030787

**Published:** 2020-01-31

**Authors:** Gazi Rahman, Hanif Sohag, Rakibul Chowdhury, Khan A. Wahid, Anh Dinh, Melissa Arcand, Sally Vail

**Affiliations:** 1 Department of Electrical and Computer Engineering, University of Saskatchewan, SK, S7N 5A9, Canada; hanif.sohag@usask.ca (H.S.); chowdhury.rakibul@usask.ca (R.C.); khan.wahid@usask.ca (K.A.W.); anv252@mail.usask.ca (A.D.); 2 Department of Soil Science, University of Saskatchewan, SK, S7N 5A8, Canada; melissa.arcand@usask.ca; 3 Research Scientist, Saskatoon Research and Development Centre, Agriculture and Agri-Food Canada, SK S7N 0X2, Canada; sally.vail@agr.gc.ca

**Keywords:** minirhizotron, root imaging, plant phenotyping, multispectral imaging

## Abstract

A minirhizotron is an in situ root imaging system that captures components of root system architecture dynamics over time. Commercial minirhizotrons are expensive, limited to white-light imaging, and often need human intervention. The implementation of a minirhizotron needs to be low cost, automated, and customizable to be effective and widely adopted. We present a newly designed root imaging system called SoilCam that addresses the above mentioned limitations. The imaging system is multi-modal, i.e., it supports both conventional white-light and multispectral imaging, with fully automated operations for long-term in-situ monitoring using wireless control and access. The system is capable of taking 360° images covering the entire area surrounding the tube. The image sensor can be customized depending on the spectral imaging requirements. The maximum achievable image quality of the system is 8 MP (Mega Pixel)/picture, which is equivalent to a 2500 DPI (dots per inch) image resolution. The length of time in the field can be extended with a rechargeable battery and solar panel connectivity. Offline image-processing software, with several image enhancement algorithms to eliminate motion blur and geometric distortion and to reconstruct the 360° panoramic view, is also presented. The system is tested in the field by imaging canola roots to show the performance advantages over commercial systems.

## 1. Introduction

Presently, rapid developments are taking place in the field of non-destructive, image analysis-based plant phenotyping, ranging from multispectral imaging to root phenotyping. A rhizotron is a non-destructive, repetitive, and belowground imaging system that can capture information on root system architecture (RSA) and root growth dynamics in situ. It also can be used to provide information needed to calculate the rates of fine-root longevity and decomposition, and to describe age cohorts of roots in relation to environmental factors [1]. Although different types of rhizotron are available, they are expensive and require technical expertise for operation and maintenance.

**Figure 1:**
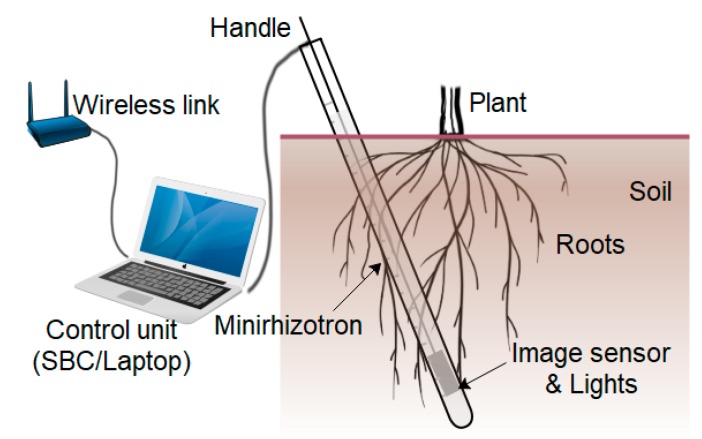
Field operation of a common minirhizotron with a wireless interface as advancement.

A minirhizotron is an alternative in situ method for viewing the roots and recording their growth through sequential photography or videography. Underground in situ root imaging is very useful for plant phenotyping research, and a minirhizotron facilitates this at a considerable convenience. Besides plant phenotyping, in situ root monitoring is becoming increasingly important in order to improve agricultural yields in a changing diverse environment. Due to its simplicity, ease of installation and low cost, it is gaining popularity. A minirhizotron consists mainly of three components: a transparent tube, a camera system with storage, and a computer as the processing unit [2,3]. The tubes are buried in the field where the underground roots are to be monitored. An illustration of a common minirhizotron is shown in Figure 1. The positioning and imaging process is mostly manual. After the images are acquired, they need to be processed for research, which is done manually or semi-automatically using customized or commercially available software. Figure 1 shows an advancement with image-storing capability both local and remotely by wireless link.

## 2. Background

Studies have shown that multispectral and/or hyperspectral root imaging improves segmentation and provides additional knowledge on physico-chemical root properties [3,4]. The work in [4] proposed various hyperspectral ranges (from 900 nm to 1685 nm) for a rhizotron system to get better contrast between different types of root and soil. However, in order for a minirhizotron to be widely adopted, its implementation needs to be low-cost, automated, and multimodal (i.e., it must support multiple imaging modalities including multispectral and hyperspectral imaging). Multimodal minirhizotron root imaging has been found useful in many cases, as described in the next section. 

Improved agricultural yields can be achieved by learning about and maintaining proper root system architecture (RSA) [5] in the field. Root-trait ranking based on RSA [6], root dynamics like feature extraction and spatial and temporal measurement [7] can assist in the understanding and improvement of RSA. Multispectral images can distinguish root tissues that differ in physiological status, i.e. living for example, (different age categories) [3,8], senescent, and dead [9], and also identify roots expressing green fluorescent protein [10]. It provides a different observational perspective of rhizosphere components, such as root tissue structure and soil minerals [11].

Correlation of nitrogen distribution in pepper plant through the leaves, stems, and roots was shown by [12] using multispectral imaging (420–1000 nm). They also grouped the wavelengths that may be used for root imaging. Nitrogen concentration was measured in wheat leaves using hyperspectral imaging (400–1000 nm) in [13]. Rhizobial infection through N-fixing noodles was monitored in [14] for legume susceptibility study for lotus root using root imaging. In another work [15], Minirizotron was used to monitor parasite growth in sunflower root using video camera in visual range. The work in [16] summarizes the use of hyperspectral imaging to detect various microbial contaminations, like bacteria and fungi in agricultural food such as, wheat, rice, and corn using TVC (Total Viable Counts). In addition, Fungal (Fusarium verticillioides) contamination development on corn kernels [17] and Aspergillus oryzae development on brown rice [18] were monitored using NIR (near-infrared) and hyperspectral imaging. The work in [19] used both hyperspectral and florescent imaging to monitor Macroalgal (epiphytic and filamentous) development in plant leaves. Moreover, multispectral imaging was used to monitor Soil carbon content (which is an important indicator of soil quality) and atmospheric CO_2_ content [20]. 

The above mentioned studies using multispectral imaging can be extended for plant roots if this modality is available to the minirhizotron platform. Therefore, in the present work, we propose a new design of a minirhizotron, known as, SoilCam, that offers multispectral imaging capability. In addition, the SoilCam can perform regular other functions of a conventional minirhizotron such as, root imaging and growth monitoring. The proposed design has been improved through several field trials. Its simplicity of operation, low power requirement, and low cost make it a good candidate for root-level plant phenotyping and soil profiling applications. In summary, the objectives of this paper are: 

(1) To automate the operation of a minirhizotron. At the same time, to provide easy and quick way to customize the minirhizotron by changing the camera, lenses, filters and light sources as per the application and need of the end user. 

(2) To extend the imaging modality in minirhizotron by adding multispectral imaging, as a possible feature and enhancement, for root-imaging and soil-profiling.

## 3. Design Objective

Considering the development cost, operational complexity, human involvement, and future use for automated root phenotyping, the following design objectives were set:

### 3.1. Tube Type

Most minirhizotron tubes presently used are made of rigid material and are 50 mm, 63.5 mm, or 100 mm in inner-diameter. There also exists an inflatable-walled minirhizotron system [21,22], but its effectiveness depends largely on continuous pressure being exerted against the soil by the flexible wall. The need for calibration of the imaging system based on the soil condition and crop type can be eliminated in a flexible-wall rhizotron tube; however, a rigid-wall rhizotron tube gives more precise image. It is also free from the pressure of a dense soil condition, and therefore preferred [23]. The tube length, buried underground, varies from 1 to 2 m. The position of the imaging unit can be adjusted by changing the depth and angular position of the tube. As shown in Table 1, minirhizotrons with 63.5 mm tubes are the most commonly used [10,24,25,26]. Based on its customizability using various image sensors, and its portability, the 63.5 mm was chosen for the design base in this study.

### 3.2. Image Sensor

The core of the minirhizotron is the imaging unit. It is essential to consider the image resolution capability because fine roots are very small in diameter and can be difficult to distinguish with low-resolution images [24]. Table 1 summarizes the imaging sensor types and their resolutions in some common commercial minirhizotrons. As seen in the table, a high-quality, crisp image of 600 DPI or more is a basic requirement to obtain the details of the roots. In addition, images should be free from geometric distortion and low-light noise, and of proper exposure. We propose to use a CMOS (Complementary Metal Oxide Semiconductor) sensor instead of a CIS (Contact Image Sensor), which will also facilitate the use of a customizable optical filter and lenses with sensors.

### 3.3. Locomotive Type

Manual imaging systems need a full-time operator to position the camera in the tube. For a common minirhizotron tube, a depth of 1m and image size per snap of 34 × 24 mm will give a total of (40 × 6) 240 pictures for full 360° coverage along the tube length. To add a 30% overlap for safety would require (58 × 8) 464 pictures. If an expert user takes 20 s for every positioning, then the total time per tube will be (464 × 20 s) 2.5 h. Therefore, it is a very tedious job for an operator to monitor the roots of two tubes per day [15,27,28]. To reduce this time significantly, a very fast, reliable, and smooth two DOF (degree of freedom) mechanical robotic system is needed. Automated camera positioning can be achieved by using geared stepper motors for better torque at a low speed with precision movements at a resolution of 0.7° along the vertical axis and 15 µm along the length of the tube.

### 3.4. Control Unit

To achieve fully automated, remote operation of the imaging system, and to control the camera positioning and imaging a high-performance 32-bit Single Board Computer (SBC) will be used.

### 3.5. Wireless Interface

Some special cases like continuous root-growth monitoring [29] demand interval imaging as frequently as every 30 min. This will generate a large quantity of image data that must be stored locally. A micro-SD card with a minimum of 32 GB will be installed. Along with local storage, this image data may also need to be stored in a remote server using a wireless link like Wi-Fi or 4G Cellular network for real-time availability. Wireless connectivity may also facilitate an internet-of-things (IoT) capability.

### 3.6. Light Source

For multispectral imaging, the type of light-emitting diode (LED)s and their brightness may need to be changed so that we can achieve proper exposure of the image. In order to facilitate this requirement, the LEDs need to be mounted in groups and their brightness will also need to be controlled separately. Two sets of LEDs will be used as the light source for the camera. All of them need to be Surface-Mount-Devices (SMDs) or Chip-On-Boards (COBs) without any lens-head in order to reduce reflection on the tube surface.

### 3.7. Power Supply

As longtime in situ and unattended operation is required for root growth monitoring [29], 24 h power backup is needed. The only commercially available, fully automatic minirhizotron (AMR-B Rhizosystems) requires 100 W [30] for its operation, and a generator as a backup power source. Therefore, to meet the requirements for longtime operation, the proposed minirhizotron needs to consume very little power. The power supply must be low cost, and maintenance free, for example, a green renewable energy source like a solar panel. The proposed SoilCam will use as little as 15 W at peak power. There will be a 12 V rechargeable gel-type lead acid battery with a solar panel and power conditioner for optimized use of the battery and solar panel.

### 3.8. System Cost

Overall the system needs to be easily affordable for multiple installations in the field. This study’s target is USD 1000 for the prototype with a basic multispectral imaging system.

## 4. Proposed SoilCam Overview

In order to meet the above design objectives, the construction of this SoilCam is divided into four main components:

1. Camera unit,
2. System control unit,
3. Power unit, and
4. Control software.

### 4.1. Camera Unit

**Figure 2:**
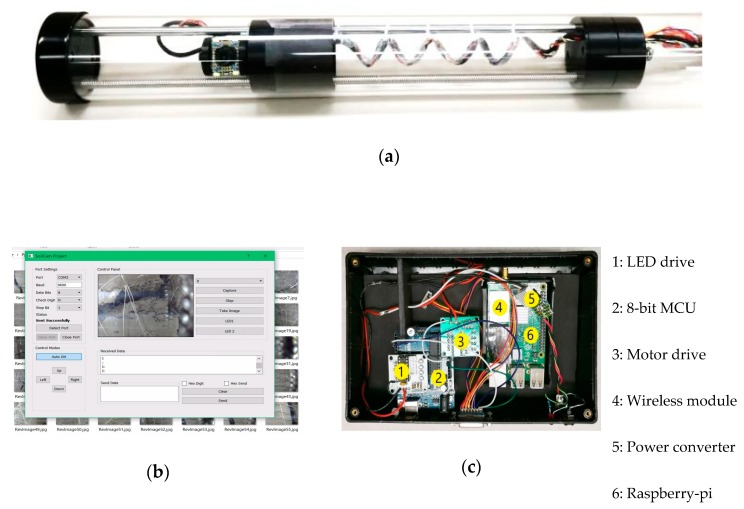
(**a**) Developed multimodal SoilCam in lab, (**b**) integrated root/soil analyzer and control software, and (**c**) control box

This unit consists of a camera and the locomotive. The camera is mounted on a 360° rotary platform that can slide through the length of the imaging tube using a lead-screw and linear bearing. Both motions are controlled using geared stepper motors. An LED assembling unit is the light-source for taking underground pictures. Figure 2a shows the camera unit with the locomotive inside the SoilCam tube. The camera can be any CMOS camera of 32 × 32 mm physical dimension along with an optional filter (IR/UV) and lens of different angle. The lighting system consists of LED of different wavelengths or colors. Both the camera and lighting system can be easily customized by the end user. 

**Figure 3:**
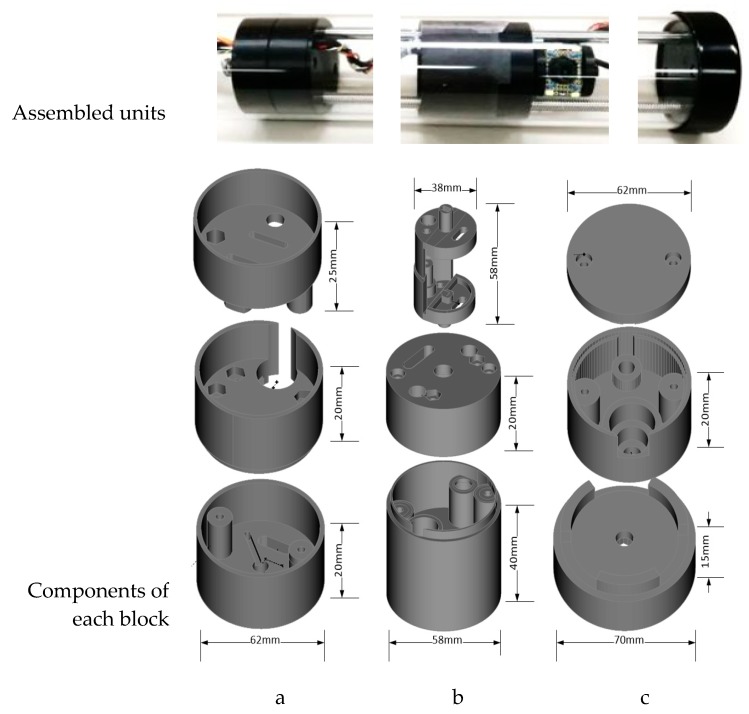
3D-printed camera mount and locomotive parts. (**a**) System base, (**b**) camera mounts, and (**c**) bottom support.

The mechanical components of the prototype were chosen with the design objectives in mind, an 8 mm guide rod with a linear bearing for smooth motion of the camera mount along the tube length is used. To control the position, a 4 × 2 mm lead-screw is used. All metal parts were made of stainless steel and brass. Bearings are also used with the lead-screw and aluminum flexible coupling for smooth rotation. For the camera rotation, there is a dedicated geared stepper motor for smooth and precise positioning of the camera. The camera rotation can be controlled at 0.7° resolution and the linear motion at 0.015 mm along the tube length. All other components, like the camera holder, mount, base and top system, are 3D designed and printed, using PLA (Polylactic Acid). All the parts have sufficient precision to a scale of 0.1 mm. Screws were leveled with the face of every part used. Figure 3 shows all the fabricated parts for the locomotive, with their dimensions. These are all 3D-printed using PLA. All the cable used inside the tube is PVC (Polyvinyl chloride) covered and twisted inside the supporting rod and the lead-screw to avoid friction with the tube body.

### 4.2. System Control Unit

**Figure 4:**
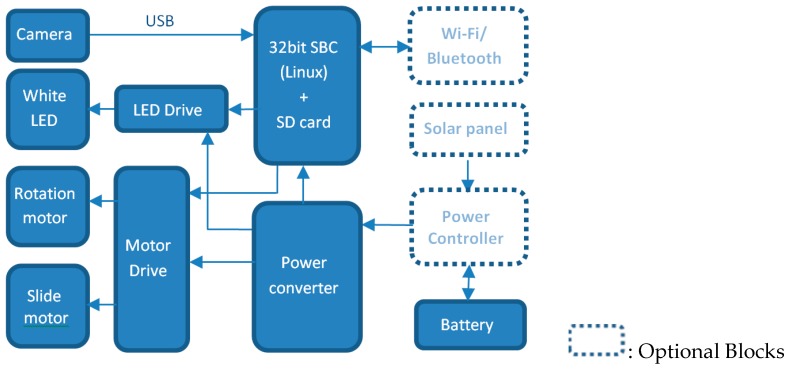
Hardware block diagram of the SoilCam.

The control system consists of two main parts - a 32-bit single board computer (SBC) as shown in Figure 4, and an 8-bit micro-controller based system as shown in Figure 2c that controls the motors and the lighting system through proper driving electronics. The software running on the SBC (or any portable Windows or Linux based devices like laptop or Raspberry-pi) gives the necessary commands to the 8-bit micro-controller to position the camera by controlling the stepper motors and to control the light source. The connections and communication flow are shown in Figure 4. The unit also controls the camera and takes and stores images using an SD Card. The main controller can also be connected to a host computer through Wi-Fi. The images stored in the SD card off-line by removing the SD card or can transfer wirelessly over the Wi-Fi. Figure 2b shows the graphical user interface of the software run in the SBC. This control unit also has all required hardware to drive the motors and control the brightness of the LEDs. Figure 2c shows the part of the control unit as used in the field trial. The motors, camera, and LEDs were chosen to meet a maximum power requirement of 15 W.

### 4.3. Power Unit

The power unit has a rechargeable 12 V lead-acid battery, solar panel, battery-charging controller and the DC–DC converter to get the necessary voltages (12 V and 5 V) for the system. This unit was designed based on the power requirement of the devices (i.e., the camera, SBC and motors) from their datasheet and the objective of unattended field operation. The power of each unit was measured in the lab and is summarized in Table 2. Then we calculated their active time of in situ operation and their required energy consumption per day was calculated (Table 2). 

Given that the user may require a complete image, 1000 mm length and 360° coverage of the tube as frequently as every 2 h, i.e., 12 full-cycle-images per day, the total daily energy requirement can be calculated as:

Total daily energy = Total energy/image x Number of images per day = 6.393 × 12 = 77 Wh.

From Table 2, the peak power of the system can also be calculated as (1.2 + 3 + 5 + 0.4 + 5) 14.6W (watt), which is in the range of the targeted design objective. Considering the total system power conversion efficiency of the power system is 80%, the total energy required per day will be (77/0.8) 96 Wh (watt-hour). As the system is powered by a battery and if the DOD (depth of discharge) of the battery is 80% and two days of autonomy are needed, then the battery and solar panel requirement can be calculated as:

Battery capacity = Daily energy x Days of autonomy / DOD Wh = 96 × 2 / 0.8 Wh = 241 Wh

Therefore, for a 12 V battery, a minimum (241 Wh/12 V) 20 Ah would be required. For the panel capacity calculation, assuming a daily charging time of 8 h and total charging efficiency of 75%, thus the required panel capacity can be calculated as:

Panel capacity = Battery capacity / (Charging Efficiency x Charge Time) = 241 Wh/(0.75 × 8) = 40W.

### 4.4. Control Software

**Figure 5:**
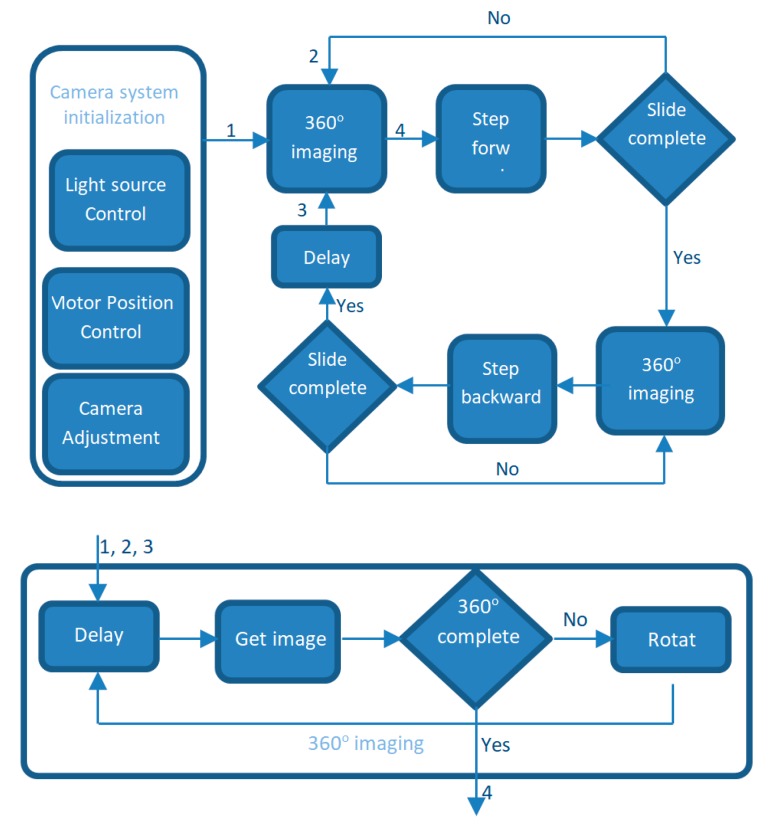
Flowchart of imaging acquisition.

The control software has three main functions: controlling the locomotive, acquiring the image, and providing a graphical user interface (GUI) for system configuration and monitoring. This software has been developed using Python-3 with Open-cv-3 and PyQt-5 libraries. It can run on a Linux or Windows based computer. The software allows the system to run automatically for a long period in a field application. The motors and LEDs are connected through the digital ports, and the camera is connected through a USB port, which is shown in Figure 4. In auto-mode, the control software captures the images, synchronizes the camera through the image-taking process, and stores the images in the SD card. Figure 5 shows the total image acquisition process in auto-mode. The GUI can also show a real-time view of the roots, making it possible to observe them without taking the device out of the tube. The intensity and on/off switches of the LEDs, can also be controlled using the software, which can turn the lights on and off individually or altogether. This feature facilitate the scope of multispectral or hyperspectral imaging of the roots.

### 4.5. System Operation

**Figure 6:**
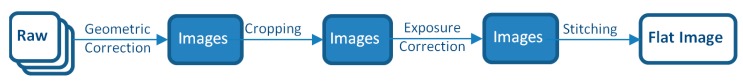
System operation steps.

This system operates in two steps: a real-time image acquisition step and an image post-processing step as shown in Figure 6. In the first step, the system takes 10 snapshots to complete a full 360° strip. Although, only six snaps are needed to cover the full 360° using a lens of 60°, an extra 40% were taken in order to get a 40% overlap for easy post-processing. In this process, the camera moves forward or backward to get the next images for 360°. For movement along the tube length, 15 mm per step will achieve at least 40% overlap between the 360° image-strips. Thus, there will be 66 strips of 10 snaps, and the total of 660 images will result in a full 1000 mm tube image. It takes 3-4 second to take 10 pictures in a 360° rotation and come back to the starting position, and around 7 min to complete the full tube length of 1000 mm. Then, post-processing algorithms are applied to reconstruct the final full-length image. Figure 6 shows the steps performed by the system to get a complete or processed image.

## 5. SoilCam in Field Trial

The first trial at a canola field was performed on 23 August, 2018, while canola was in its late pod-filling to mature stage. The data collected consisted of 231 images from 21 strips (a complete 360° image) of 11 snaps each, covering 320 mm of the tube length. These images then were processed in the lab. As a result several problems were observed, namely:

1. Motion blur along the direction of the camera rotation;
2. Angular shift among the strips due to mechanical displacement of the rotating camera mount;
3. Geometrical distortion due to the wide-angle lens;
4. Geometrical distortion and focus problems due to the cylindrical surface of the imaging object, i.e. minirhizotron tube; and
5. Marks of light reflection in the images mainly on the tube surface and the shiny mechanical parts

Problems 1 and 2 were removed fully by modifying the mechanical design. The rest were removed and/or improved using image post-processing algorithms as described in Section 5.2.

### 5.1. Modifying the Mechanical Design

**Figure 7:**
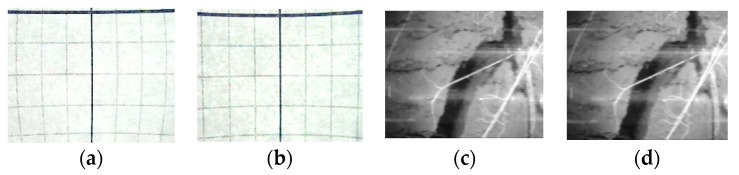
Applying Barrel distortion correction: (**a**) distortion profile, (**b**) corrected profile; (**c**) distorted image, and (**d**) corrected image after application.

The motion-blur problem was solved by introducing a controllable delay, using the control software while taking the images. The camera was placed on a rigid support to overcome the mechanical displacement of the camera mount. The focus problem was solved by changing the camera lens to one of better quality and narrower angle. Light-source reflection was removed by using continuous COB (Chip on Board) LEDs as the light source and relocating the lead-screw-guide rod arrangements. In this design, the facility to replace the LEDs, camera lens, and filter easily was introduced in order to acquire multispectral images. The geometrical distortion profile was measured for the camera, lens, and the cylindrical surface of the minirhizotron tube in the lab, and the same corrective profile was applied for the correction of the actual images collected in the field, Figure 7a,b show the original distortion profile graph and the corrected graph respectively, after the correction algorithm [31] is applied. The same algorithm is applied on the acquired root image and shown in Figure 7c,d. The measures described below were also taken to reduce these distortions in the next field test, and thus, for further use of the system.

### 5.2. Image Post-Processing.

**Figure 8:**
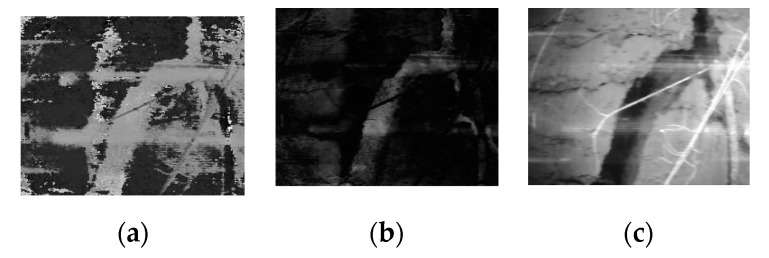
Division of image in HSV plane: (**a**) H Plane, (**b**) S Plane, and (**c**) V Plane.

In order to remove reflection of the indirect light, i.e., the light reflected from the internal surface of the tube, several correction algorithms in MATLAB were applied. First, the reflection marks of the light source on the images needed to be removed. A close inspection of the raw image in the HSV (hue, saturation, value) plane showed that most of the reflection resided in the Hue channel of the image. The division of the original image into the HSV plane is shown in Figure 8. A thresholding technique applied to the Hue plane removed the reflection to a great extent. After thresholding the H (hue) plane, all the images were rejoined and converted to the RGB (red, green, blue) image again. 

After the reflection marks were removed, the following post-processing techniques were applied:

1. Geometric correction (wide-angle lens distortion, tubular surface imaging distortion);
2. Image normalization (color correction, exposure correction);
3. Image crop (extraction of the usable non-overlapped images); and
4. Image stitching to get a complete 360° strip of the root image.

**Figure 9:**
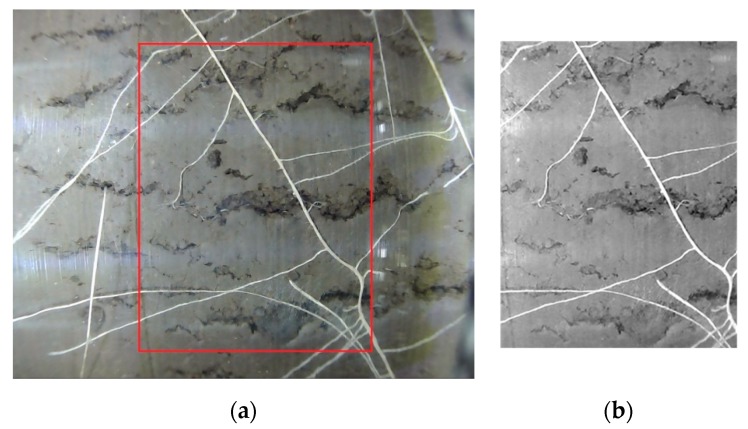
(**a**) Image before processing and (**b**) image after processing (cropped).

**Figure 10:**
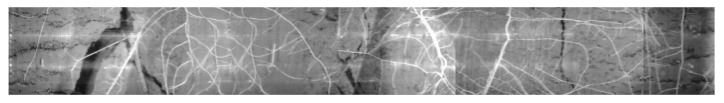
A 360° view of canola root taken by SoilCam (after stitching 10 images together).

After the above image post-processing tasks were performed, a sharp and undistorted image was obtained, as shown in Figure 9. Figure 10 shows a complete 360° view of canola root after post-processing and stitching of 10 images. Image processing was applied (as shown in Figure 7 and Figure 8) using MATLAB. It took 2.683 s including image loading for a single image using an Intel core-i7 computer with 8 GB RAM and running Windows 10-64 bit. 97% of the total time was taken for the correction of geometric distortion. Therefore, the total time to process 660 images to complete a 360° × 1 m (before stitching) image is around 30 min.

### 5.3. Multispectral Image Acquisition

**Figure 11:**
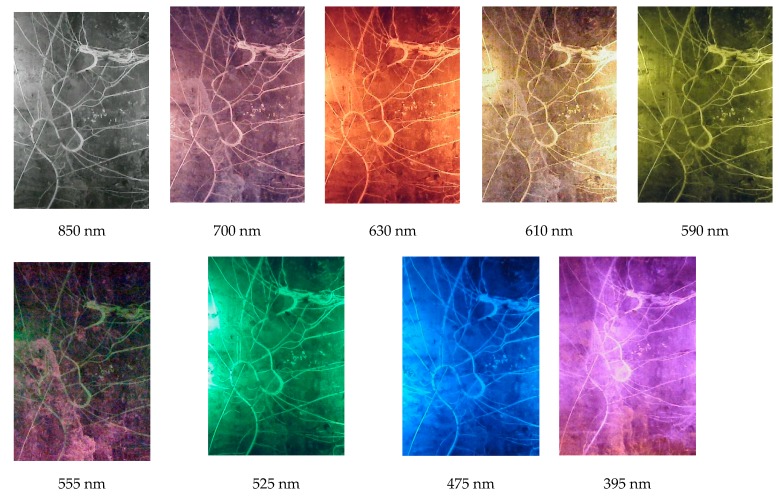
Multispectral root images at various wavelengths.

After the redesign of the system, the second field trial was performed on 26 September, 2018. This time multispectral images were acquired by using LEDs of various wavelengths, changing the lens and removing the IR filter of the camera. The images of the roots were captured on the same day using the same orientation for comparison. Figure 11 shows some of the multispectral images. The wavelengths used are 850 nm, 700 nm, 630 nm, 610 nm, 590 nm, 555 nm, 525 nm, 475 nm, 465 nm, and 395 nm using LEDS of different wavelengths. 

## 6. System Performance

The lab prototype was designed for a tube of 63.5 mm inner diameter and 500 mm in length to make it easy to test in a lab environment and for a quick field trial. After some design improvements, as described in Section 5.1, all of the targeted design objectives were achieved. The light source, camera, filter, and lens were easily replaceable for multispectral imaging, and for improved DPI. All mechanical moving parts were fully enclosed and protected from dust and humidity. The mechanical compartment had enough lubricating fluid and the special cleaning fiber kept the tube surface clean while moving. The software worked properly and the camera position could be aligned precisely. Images were captured both manually and automatically through 360° and the length of the tube, and the light sources were controlled by the software user interface (UI). The technical specifications are summarized in Table 3. 

## 7. Comparison

There are some commercial minirhizotron imaging systems with various features available in the market. Most of them are manual or semi-automatic in operation. With the semi-automatic systems, radial/angular positioning is done by the system itself, so the user needs to only do the vertical positioning, using manual indexing. The CI-600 takes around 480 s per rotation while scanning the tube radially at 600 DPI [26]. While it can give an image of 196 mm vertical length, the user needs to reposition the imaging unit six times along the length of a 1 m tube with a 15% overlap. Thus for 600 DPI, the total time required for one tube is 6 x 480 s = 48 min and this time may vary with the image quality from 100 DPI to 1200 DPI (for CI-602). 

Most matrix-type CMOS image sensors provide images of 34 × 24mm, therefore, one image covers a small portion of the total minirhizotron tube area. These images also suffer from geometric and non-uniform focus problems [26]. Users may need to correct these errors using software in order to use them. To get a complete image from the acquired images, it may require further post-processing, which demands a significant amount of time. Using some automated software this can be minimized, however, it also needs some manual effort. The imaging system based on CIS can overcome the geometric and focusing distortion by its scanning operation. However, we faced difficulty to insert the CI-600 system in some tubes due to bending, which was not the case for the designed prototype. A matrix image sensor based camera can facilitate continuous imaging throughout the operation of image acquisition, which can be used to get the image of any position instantly. It can reposition or register the camera (using the software running in the SBC) easily in the tube, or can acquire the image again at higher DPI. However, the CIS needs to complete a full scanning to give an image and which is very time consuming to do any re-positioning or re-taking an image at higher DPI.

The AMR-B of Rhizosystems is the only model that accommodates auto-registration and a rotation feature. However, it is only available for a 100 mm tube and requires almost 100 W of electric power, which makes it impractical for lengthy field operations with stand-alone power sources. It also requires a significant amount of maintenance.

The powerful and dedicated control unit, single board computer, along with the control software of the SoilCam can facilitate long-time automated operation without any human interaction. The solar backup also can facilitate lengthy field operations. With wireless data acquisition, a user can control the system and transfer the images to a host computer for offline data processing without removing the imaging system from the field. According to our field trial, we found that the average size of an image file is 310 k B using the 2 MP camera. Therefore, for a 360° image and 1 m tube length, we will need 660 images of 205 MB of data. Using a 3G wireless link with a data transfer rate of 3 Mbps and 10% data transfer over-head, it will take (205 × 8 × 1.1/3) 600 s. Therefore, it will take 10 min to transfer the data for a 360° x 1 m image. Since the camera unit of the proposed system can be replaced easily, image resolution can be increased as desired. The imaging area also can be expanded by increasing the length of the camera mount and the sliding system. By changing the lens, the camera system can be made narrower and lighter, and it will also help to insert the system in a reasonably bent tube. In addition, this system can be used as a scanner to generate underground video, unlike the CIS system which can only produce still images. 

Compared with available commercial systems, the proposed system has some inherent advantages. The available minirhizotron solutions cost in the range of USD 16,000 to USD 25,000, whereas the cost of this prototype is around USD 650. A detailed cost breakdown is shown in Table 4. For a full 1000-mm long imaging system, wireless interfacing, and a solar panel, the cost may increase maximum of USD 350 for a total of USD 1000. For this low cost, several units of the proposed system can be installed simultaneously for the same cost as a single unit of a present commercial system. As this would be an inexpensive solution for the researcher to use a single SoilCam to take images from multiple different locations within a single field experiment, 20 tubes as an example at a much lower cost than the existing solution. Hence, it would be more affordable to be used for various researches, to have enough images across multiple experimental units to have some measure of variability, with multiple different experimental treatments, this can magnify the number of separate tubes that would be required. So, the SoilCam has many advantages over the other commercial systems including costs, it doesn’t completely solve the problem of needing multiple tubes to be imaged. However, it is still probably cheaper to have four cameras do the work that only one commercial could do, and with the added value of multispectral images.

Other commercial models like BTC-2 and BTC-100x are presently not in production [32]. There have been several minirhizotrons imaging systems developed and operated as lab prototypes only [24,33,34,35,36]. A summary of the comparison among the commercially available systems is shown in Table 5. 

## 8. Conclusions

This study has proposed a low-cost and fully automated minirhizotron system for use in a real-time root monitoring application. This system, called SoilCam, uses commercial off-the-shelf components, and is the most cost effective solution compared with available commercial systems. To the best of our knowledge, it is the only minirhizotron system that can capture multispectral images of crop roots in situ. Due to its low cost and ease of manufacturing, it can be used widely in both research and commercial crop root monitoring. Because it is fully automated and consumes very little power, it can be installed in the field for long periods of time and continuously takes images of roots without any human involvement, thus, reducing the total operational cost of the system. In addition to imaging, various sensors, like temperature, pH [38], and gas sensors, can provide further information on soil properties and are also helpful in determining soil characteristics by effectively identifying soil organic matter separately from minerals [39]. Underground CO_2_ efflux [23], and for the study of concentration of soil bacteria [40] in response to environmental changes of CO_2_ and O_3_, minirhizotron, can also be a useful tool for in situ and concurrent imaging. As the software is very simple and can be used both manually and automatically, users can easily employ it to take customized images. It can also generate time-lapse video clips, allowing easy observation of root growth. Moreover, the design of the system attempts to overcome the limitations of existing commercial systems, while also requiring less maintenance than previous systems. However, there are still some areas for future improvement, such as local image processing, power optimization, high-speed imaging, and lower cycle times, which will be addressed in future design cycles. 

## Figures and Tables

**Table 1:** Imaging techniques and tube diameters in commercial minirhizotrons.

Model	CI-600	CI-602	MS-16/17	AMR-B	Manual MR
Imaging unit	CIS *	CIS	CMOS *	CMOS	CMOS
Resolution (DPI)	100–600		2500	300	1900
Tube diameter (mm)	63.5	50	63.5	100	50
Manufacturer	CID Bio-science		Vienna Scientific Instruments	RhizoSystems, LLC	

* CIS: Contact Image Sensor, CMOS: Complementary Metal Oxide Semiconductor.

**Table 2:** Component energy requirement per cycle.

Component	Power (W)	Active time (s)	Total Energy (Wh)
Camera	1.2	2178	0.726
Motor-1	3	1980	1.65
Motor-2	5	198	0.275
Light source	0.4	2178	0.242
Controller & SBC ^1^	1.75	7200	3.5
Total energy requirement per image			6.393

^1^ Average power was found for a complete imaging cycle is 1.5 W. Its peak power is 5 W.

**Table 3:** Specification of the prototype of the proposed SoilCam.

Criteria	Range
Camera type	2MP CMOS sensor 170° Wide angle lens, no optical filter
Image size	35 × 28mm (1400 DPI)
Camera rotation	360°
Camera system length	550 mm
Camera system maximum diameter	62 mm
Imaging length	330 mm
Flat image area coverage	200 mm x 330 mm
Camera system power	12 V, 10 W (peak)
Controller power	12 V, 5 W (peak)
Control unit	32 bit SBC
Power source	12 V 20 Ah Gel battery
Operation duration	24 h @ 60 min imaging interval
Optional power	40 W peak solar panel (not used)

**Table 4:** Cost detail for the first prototype of the proposed system (solar backup system is not included).

Item	Model/Part Number	Quantity	Cost (USD)
8 mm, 500 mm lead-screw and support rod set	T8-500	1 set	170
Camera (2 MP) module	ELP-USBFHD04H-L170	1 pc	80
Geared stepper motor	28BYJ-48	2 pcs	15
Motor drive	ULN2003	2 pcs	10
3D-printed parts (10 pieces)		1 Set	100
32-bit SBC	Raspberry-Pi 3B	1 pc	85
8-bit Controller	ATMega-328 board	1 pc	15
Light sources	LEDs		25
Other electronics, cables, and connectors		As needed	75
Battery	12 V, 6 AH	1 pc	25
Housing and hardware		As needed	50
Total			650

**Table 5:** Comparison of main features among the commercial, lab-prototype, and proposed system.

Model (Commercial)/Name (Prototype)	Image Resolution (DPI)	Imaging Technique	Live Imaging	Illumination Control	Control Mode	Usable Tube Inner Diameter	Tube Length	In Situ Operation	Power Requirement
CI-600 (CI-602) [26]	100–600 (600–1200)	RGB-CIS	Not Possible	None	Auto 360°, Manual height	63.5 mm (50 mm)	100 cm	Need Human interaction	Low
VSI MS-190 [32]	2500	RGB CMOS	Not available	UI adjustable brightness	Semi-Auto 360°, Manual height	70 mm, 60 mm	100–200 cm		Medium
AMR-B [30]	300			None	Auto 360° Auto height	110 mm	100 cm		High (110W)
Manual MR [37]	1900			None	Manual 360°, Manual height	50 mm	100, 200 cm		Low
SoilCam (Proposed)	600-2500+	Multi/Hyper-spectral	Yes	UI adjustable brightness	Auto 360°, Auto/ Manual height	63.5 mm, 110 mm	100–200 cm	No human interaction required	Low (10W+ solar panel)
Marc Faget, 2010 [10]	480 **	NIR	Yes	Fixed	Manual	63.5 mm	100 cm	Need Human interaction	Medium
M. Amato, 2012 [24]	480 *	USB Microscope	Yes	None	Manual	63.5 mm	200 cm		Medium
Randy T. C, 2011 [38]	2893 ***	DSLR *1	No	Manual	Manual	2D Flat Bed			Low

* Dino-Lite AM3111 0.3MP Digital Microscope; ** Creative Live Cam Notebook Pro; *** Nikon D200.
